# Evolutionary Connectionism: Algorithmic Principles Underlying the Evolution of Biological Organisation in Evo-Devo, Evo-Eco and Evolutionary Transitions

**DOI:** 10.1007/s11692-015-9358-z

**Published:** 2015-12-08

**Authors:** Richard A. Watson, Rob Mills, C. L. Buckley, Kostas Kouvaris, Adam Jackson, Simon T. Powers, Chris Cox, Simon Tudge, Adam Davies, Loizos Kounios, Daniel Power

**Affiliations:** 1 Agents, Interactions and Complexity, ECS, University of Southampton, Southampton, UK; 2 Institute for Life Sciences, University of Southampton, Southampton, UK; 3 Biosystems & Integrative Sciences Institute (BioISI), Faculty of Sciences, University of Lisbon, Lisbon, Portugal; 4 School of Engineering and Informatics, University of Sussex, Falmer, UK; 5 University of Lausanne, Lausanne, Switzerland

**Keywords:** Evolutionary developmental biology, Evolutionary ecology, Major transitions in evolution, Learning theory

## Abstract

The mechanisms of variation, selection and inheritance, on which evolution by natural selection depends, are not fixed over evolutionary time. Current evolutionary biology is increasingly focussed on understanding how the evolution of developmental organisations modifies the distribution of phenotypic variation, the evolution of ecological relationships modifies the selective environment, and the evolution of reproductive relationships modifies the heritability of the evolutionary unit. The major transitions in evolution, in particular, involve radical changes in developmental, ecological and reproductive organisations that instantiate variation, selection and inheritance at a higher level of biological organisation. However, current evolutionary theory is poorly equipped to describe how these organisations change over evolutionary time and especially how that results in adaptive complexes at successive scales of organisation (the key problem is that evolution is self-referential, i.e. the products of evolution change the parameters of the evolutionary process). Here we first reinterpret the central open questions in these domains from a perspective that emphasises the common underlying themes. We then synthesise the findings from a developing body of work that is building a new theoretical approach to these questions by converting well-understood theory and results from models of cognitive learning. Specifically, connectionist models of memory and learning demonstrate how simple incremental mechanisms, adjusting the relationships between individually-simple components, can produce organisations that exhibit complex system-level behaviours and improve the adaptive capabilities of the system. We use the term “evolutionary connectionism” to recognise that, by functionally equivalent processes, natural selection acting on the relationships within and between evolutionary entities can result in organisations that produce complex system-level behaviours in evolutionary systems and modify the adaptive capabilities of natural selection over time. We review the evidence supporting the functional equivalences between the domains of learning and of evolution, and discuss the potential for this to resolve conceptual problems in our understanding of the evolution of developmental, ecological and reproductive organisations and, in particular, the major evolutionary transitions.

## The Evolution of Developmental, Ecological and Reproductive Organisations

### The Problem: Variation, Selection and Heredity are Modified by Organisations that are Themselves Evolved

Evolution by natural selection aims to explain biological adaptations—such as how the giraffe came to have a long neck. For some purposes it is sufficient to assume that there is, for example, variability in neck length, that long necks confer differential survival or reproductive benefit, and that neck length is heritable. However, research in the rapidly-expanding fields of evolutionary developmental biology (evo-devo), evolutionary ecology (evo-eco) and the evolutionary transitions in individuality (for which we propose the term “evo-ego”, Watson and Szathmáry 2015) recognises that for some purposes this is only part of the explanation. That is, evolutionary outcomes are significantly dependent on how developmental, ecological and reproductive organisations constrain or facilitate the variability of phenotypes, the organisation of their selective environment and the heritability of the relevant evolutionary units, respectively. For example, how is it that developmental constraints allow multiple phenotypic traits to change simultaneously whilst maintaining suitable functional integration between them and avoiding deleterious side-effects on other traits? What is it about the organisation of an ecological community that causes some ecological relationships to remain stable over long periods of selection and applies a strong selective pressure for changes in other ecological relationships (e.g. between a particular herbivore and a particular resource)? How is it that reproductive constraints (e.g. reproduction through a single-celled population bottle-neck) come to define a multicellular organism like a giraffe as a Darwinian unit in the first place—suppressing fitness differences between the cells or genes within an individual (so they do not compete with each other for representation in offspring individuals) but enabling the inheritance of fitness differences between individuals (allowing them to compete for representation in the population)? Answering these questions requires a move to a different level of explanation—one that attempts to explain why variation, selection and inheritance have the forms that they do, rather than taking them as fixed axioms of the adaptive process.

These parameters are not simply exogenous contextual details to the processes of evolution. Each of these organisations (developmental, ecological and reproductive interactions) is itself a product of evolution or is modified by the products of evolution (Wagner and Altenberg 1996; Odling-Smee et al. 2003, Okasha 2006; Laland et al. 2011, 2015). The phenotypic variation exhibited by a genetic lineage is modified by the evolution of developmental interactions (Brakefield 2006; Kirchner and Gerhart 1998; Wagner and Altenberg 1996; Toussaint and von Seelen 2007), the selection it experiences is modified by the evolution of ecological interactions (Post and Palkovacs 2009; Laland and Sterelny 2006; Laland et al. 1999; 2011; Odling-Smee et al. 2003, 2013), and even the identity of the evolutionary unit changes as a result of the evolution of new reproductive strategies and new mechanisms of inheritance (Jablonka and Szathmáry 1995; Szathmáry and Demeter 1987; Okasha 2006; Sigmund and Szathmáry 1998; Maynard Smith and Szathmáry 1995). Accordingly, each of the major components of the Darwinian machine (namely, variation, selection and inheritance) (Lewontin 1970) are themselves subject to evolutionary change (Watson and Szathmáry 2015). The ambitious aim of an extended evolutionary synthesis (Pigliucci and Muller 2010; Laland et al. 2015) can thus be seen as the aim to endogenise these organisations into evolutionary theory (Okasha 2006, p. 220)—explaining both how evolutionary processes shape these organisations (evo → devo/eco/ego) and, in the reverse direction, how these organisations affect evolutionary outcomes (evo ← devo/eco/ego). This is not easy to do (Pigliucci 2007; Lawton 1999; Laland et al. 2011, 2015; Okasha 2006).

*In evolutionary developmental biology (evo-devo)* (Brakefield 2006; Carroll 2008; Wagner and Laubichler 2004; Wagner et al. 2007; Wagner 2013; Hoekstra and Coyne 2007) it is recognised that the organisation of developmental biases and constraints controls the distribution of phenotypic variation that is produced under genetic (or environmental) variation and can thereby control the possible paths of evolutionary trajectories through phenotype space (Arnold et al. 2001; Jones et al. 2007; Schluter 1996; Toussaint and von Seelen 2007; Gerhart and Kirschner 2007; Wagner 2014) (evo ← devo). For example, some physiological changes in limb morphology, wing patterns or gene-regulatory circuits may be readily produced and selected whereas others may not (Wagner 2014; Brakefield 2006). The structure of this developmental organisation is itself subject to change over evolutionary time, e.g. via the evolution of gene-regulatory interactions or morphological architectures (Riedl 1977; Draghi and Wagner 2009; Wagner and Altenberg 1996; Crombach and Hogeweg 2008; Amundson 2005; Pavličev and Cheverud 2015) (evo → devo). This bi-directional interaction means that evolution could modify developmental organisation in a way that facilitates or frustrates future evolution—hence the *evolution of evolvability*, i.e. evolved changes that affect the future ability of a population or lineage to evolve (Wagner and Altenberg 1996; Kirchner and Gerhart 1998; Chicurel 2001; Partridge and Barton 2000; Hendrikse et al. 2007). Work on this topic shows that the evolution of gene-regulatory interactions or phenotypic correlations can enhance phenotypic robustness (Wagner 2008, 2013), accelerate adaptation under directional selection (Pavlicev et al. 2011), or evolve to mimic the structure of the selective environment (Watson et al. 2014; Riedl 1977), such as modularity (Lipson et al. 2002; Watson et al. 2014; Clune et al. 2013; Kashtan et al. 2007, 2009; Parter et al. 2008). But the general relationship between the evolution of individual developmental interactions and developmental organisations, and in particular the evolution of structures that facilitate evolvability, remains poorly understood and conceptually problematic (Pigliucci 2007; Chicurel 2001; Partridge and Barton 2000; Sniegowski and Murphy 2006; Hendrikse et al. 2007). In particular, the idea that natural selection might change the variability on which it depends introduces a self-referential element that is particularly difficult to characterise with conventional theory.

*In evolutionary ecology (evo-eco)* (Matthews et al. 2011; Post and Palkovacs 2009; Schoener 2011; Müller 2007), the details of the relationships in an ecological community have a significant effect on the selective pressures acting on component species (evo ← eco). For example, the prevalence of a particular resource or competitor may be strongly influenced by the existing ecological relationships in the community, and may also dominate the selective pressures on an evolving population within that community. The structure of these ecological organisations is also itself modified by the changing nature of ecological relationships, due to the evolution and coevolution of the component species in interaction with one another (e.g. changes in individual characters that modify the overlap of resource utilisation profiles, or by traits that modify the energy, time or resources invested in exploiting one ecological relationship rather than another) (evo → eco)—see *niche construction* and *environment engineering* (Odling-Smee et al. 2003; Laland et al. 1999; Wright and Jones 2006; Post and Palkovacs 2009). The organisation of ecological relationships (both trophic and non-trophic) can affect the stability, resilience and homeostatic dynamics of ecological communities and complex ecological functions (Jax 2010; Lenton 2004; Holling 1973; Folke 2006; Gallopín 2006). Some argue that the reciprocal causation involved in evo-ecological interactions (i.e. evolution occurs in an ecological niche and the ecological niche is itself a product of evolutionary processes; Watson and Ebner 2014; Post and Palkovacs 2009) constitutes a significant departure from conventional evolutionary models (Laland et al. 2011; Mesoudi et al. 2013). But the general relationship between the evolution of individual ecological relationships and community organisation, and in particular the evolution of ecological feedbacks that facilitate self-regulation or homeostasis, remains poorly understood and conceptually problematic (Lawton 1999; Cropp and Gabric 2002; Okasha 2005; Lenton and van Oijen 2002). In particular, the idea that natural selection might change the selection pressures that act on itself introduces a self-referential element that is difficult to characterise with conventional theory.

*In the major evolutionary transitions (evo-ego)* (Maynard Smith and Szathmáry 1995, Godfrey-Smith 2009; Michod 1999, 2007; Bourke 2011; Buss 1987; Okasha 2006; Bouchard and Huneman 2013), evolution has repeatedly reinvented itself—creating new evolutionary units at successive scales of biological organisation, e.g. from self-replicating molecules, to chromosomes, to simple cells, to multi-organelle eukaryote cells, to multicellular organisms, to eusocial groups. These are not just changes in the characteristics of an existing evolutionary entity, but the result of changes to the reproductive relationships between evolutionary entities such that “entities that were capable of independent replication before the transition can replicate only as part of a larger whole after the transition” (Maynard Smith and Szathmáry 1995). These changes, i.e. changes that prevent independent replication, act to suppress fitness differences between individuals at one level of organisation and may provide opportunities for natural selection to create heritable fitness differences at a higher-level of organisation (Godfrey-Smith 2009; Okasha 2006; Michod and Roze 2001; Michod and Herron 2006; Ryan et al. 2015). This may involve reproductive organisations that synchronise the transmission of information across generations (e.g. vertical transmission of symbionts, Margulis and Fester 1991; compartmentalisation of replicators, Sigmund and Szathmáry 1998; Szathmáry and Demeter 1987; or linkage of replicating molecules into chromosomes, Maynard Smith and Szathmáry 1993) or restrict the channels of communication (e.g., bottle-necked life-cycle, germ-soma separation; Godfrey-Smith 2009; Buss 1987). Such features change the scale at which heritable variation in reproductive success is manifest, thus resulting in the evolution of new levels of Darwinian individuality (Maynard Smith and Szathmáry 1995; Jablonka 1994; Clarke 2010; Okasha 2006; Godfrey-Smith 2009; Wilson 1989; Bouchard and Huneman 2013) (hence, “evo-ego”). Note that the evolutionary unit can be defined by the level (or levels) of organisation where variation in reproductive success is heritable (evo ← ego) (Clarke 2010; Godfrey-Smith 2009), and the level of organisation that exhibits this can be modified by the products of the evolutionary process (evo → ego). Work to understand these evolutionary transitions in individuality is motivating a new research programme within evolutionary biology (Calcott and Sterelny 2011; Okasha 2006, Bouchard and Huneman 2013) that encompasses concepts such as the *evolution of individuality*, *social group transformation*, *de*-*Darwinisation* (of individuals) and *Darwinisation* (of groups) and *export of fitness* (from lower to higher units) (Buss 1987; Bourke 2011; Godfrey-Smith 2009; Okasha 2006; Michod 1999). But at present the organising principles involved in the evolution of reproductive dependencies, and in particular the evolution of new levels of evolutionary individuality, remain poorly understood and conceptually challenging (Okasha 2006; Godfrey-Smith 2009). In particular, the idea that natural selection might redefine the evolutionary unit, i.e. the reproductive heritability on which it depends, introduces a self-referential element that is difficult to characterise with conventional theory.

Each of these areas shares the common underlying conceptual problem caused by bi-directional interactions or reciprocal causation (Levins and Lewontin 1987; Post and Palkovacs 2009; Laland et al. 2011, 2015), i.e. evolution modifies organisations (often naturally described as networks) and these organisations modify the process of evolution. The notion that evolutionary processes can thereby change their own parameters is the root cause of theoretical and conceptual roadblocks in each field (Watson and Szathmáry 2015).

In particular, although it is clear that evolution can modify the parameters of variation, selection and inheritance, and this *might* facilitate evolvability, it is not at all clear that these organisations can be adaptations *for* increased evolvability. In the absence of a higher-level selective process that might favour evolutionary processes that are successful in the long term, it seems equally likely that such organisations might constrain or frustrate subsequent adaptation. For example, researchers in evo-devo struggle to agree on whether the long term evolvability of a population can systematically improve as a consequence of natural selection acting on developmental organisations (Pigliucci 2008; Sniegowski and Murphy 2006; Chicurel 2001; Partridge and Barton 2000; Pavlicev et al. 2011; Earl and Deem 2004). Evo-eco recognises the pressing need to predict how ecological resilience and ecosystem functions change over time (Holling 1973; Gallopín 2006; Jax 2010). Although evolution might change community organisation in a way that increases the resilience or self-regulation of the ecosystem (Cropp and Gabric 2002), when there is no selection at the ecosystem level it seems equally likely to become more susceptible to stresses and perturbations over evolutionary time, possibly resulting in catastrophic collapse in the long term (Holling and Gunderson 2002; Montoya et al. 2006; Schoener 2011). Work in evo-ego seeks to understand whether evolutionary transitions in individuality result from systematic adaptive pressures toward the creation of higher-level biological organisations or whether they are merely a collection of independent accidents (Maynard Smith and Szathmáry 1995; Okasha 2006). It might be the case that the reproductive dependencies evolved at one level of organisation create higher-level organisations that are better-able to respond to selective pressures relevant at the higher level of organisation, but alternatively it might be the case that they encapsulate maladaptive relationships that oppose an effective response to higher-level selection.

Such problems motivate the growing recognition that the Darwinian machine needs an overhaul: That self-referential evolutionary mechanisms (where the products of evolution alter the processes of evolution) create serious problems for existing theory (Laland et al. 2011), and that a new and expanded theoretical framework is needed (Pigliucci and Muller 2010; Laland et al. 2015) that integrates “eco-evo-devo” processes (Blute 2008) (and we would add “ego” to this list also).

The same underlying problem of reciprocal causation is manifested differently in each domain. Whilst it is clear that the products of the Darwinian machine can modify the parameters of its own operation, it is not clear in what way it changes itself and, in particular, whether it is possible that the Darwinian machine changes systematically ‘for the better’, i.e. in a way that facilitates rather than frustrates subsequent adaptation. This problem arises in domain-specific versions:

- Evo-devo—implications for modifying variability, and the evolution of long-term evolvability:
*Can development be organised to facilitate future adaptation*? That is, can evolution ‘predict’ or ‘anticipate’ what developmental organisations will enable adaptive variation in new environments that it has not yet been exposed to? Conversely, if all it does is find organisations that are fit over the set of past environments, then that seems to be conventional evolution, not the evolution of evolvability.
- Evo-eco—implications for modifying the selective context, and the evolution of ecosystem organisation:
*Can an ecosystem be organised ‘for’ anything if it is not an evolutionary unit*? That is, how can natural selection at the level of individuals within multiple species result in ecological organisations that are self-supporting at the system level (homeostasis), or indeed, result in any kind of ‘ecosystem evolution’ that is more than the sum of the evolution of the parts? (Levin 2011; Leigh and Vermeij 2002; Lenton 2004). It is clear that by evolving its ecological relationships a species may modify the ecological dynamics of the community and hence it’s ecological context and hence the selection it experiences over subsequent generations (i.e. niche construction, Odling-Smee et al. 2003, 2013). But it is not clear that it can do this in a way which is systematically beneficial to itself, except in the case where such benefits fall differentially on the individual bearing the niche constructing trait (which implies it can be treated as an extended phenotype of the individual). For example, an individual character that reduces competition for resources with another species offers no differential advantage to the trait bearer if all members of its species benefit from such reduced competition (Wilson 1980). Thus we may expect that the network of ecological relationships that evolve may alter ecological dynamics and attractors, but not necessarily in a manner that creates adaptive benefits to the species, let alone the community as a whole.
  To clarify, we use the term ‘ecosystem evolution’ to refer to the ecosystem-level consequences of natural selection acting on individuals within component species (Levin 2011, 2014), e.g., evolutionary change in the inter-specific relationships of an ecological community arising from individual-level selection. We do not suggest that ecosystems or ecological communities are units of selection (which would require a population of multiple ecosystems with heritable characteristics). Nonetheless, we ask whether there exist conditions where individual selection has emergent yet predictable consequences for the organisation and efficiency of the system as a whole (Levin 2011). Such questions are related to questions regarding collective welfare in economic systems under the assumption of individual utility-maximising agents (Levin 2014), and the reciprocal interaction of social behaviour with co-constructed social structures, e.g. institutions, a.k.a. *the agency*-*structure debate*, (Bator 1957, Ritzer and Goodman 2004).
- Evo-ego—implications for modifying heritability, and the evolution of new evolutionary units:
*Can evolution at one level of organisation favour the creation of heritable evolutionary units that are adaptive at a higher-level of organisation*? That is, can the evolution of reproductive organisations find new heritable units that are suitable for responding to selection at the higher level of organisation before that level of organisation exists? (Trestman 2013). Like the previous question, this is related to the formation of social structures that change individual incentives. But the outcome in this case is even more radical—not merely the evolution of interaction structures that incentivise cooperation in individuals, but the evolution of reproductive dependencies that create a new evolutionary unit, subsuming the Darwinian individuality of the original evolutionary units. Moreover, not only do we ask whether individual selection can create new evolutionary units, but whether the new units it creates are effective at facilitating adaptation at a higher level of organisation—or conversely, whether they frustrate further adaptation.

In each case, the conventional answer seems to be—*it cannot*. It is not possible for evolution by natural selection to produce adaptations for an environment it has not yet encountered, to produce organisation at the system level without selection at the system level, or to create new units that are adaptive for a level of selection that does not yet exist.

### Well-Understood Solutions in Learning Systems

We present the case that all of these behaviours that seem impossible for evolutionary systems are possible, and that necessary and sufficient conditions can be characterised. We make this argument by recognising that analogous behaviours are possible, and are well-understood, in another domain—and because the underlying principles are mathematically equivalent, specific results and insights from one domain can be transferred to the other (Watson and Szathmáry 2015). This is a domain where the idea of a system that changes itself over time is not controversial—namely, *learning systems*.

A learning system is a system that improves its performance at some task with experience (Mitchell 1997). A simple kind of learning (often likened to natural selection) is *reinforcement learning*. This utilises a reward function to reinforce good behaviour or good outputs (or punish bad outputs) when they occur. An analogy between this type of learning and evolution by natural selection is common and intuitive (Maynard Smith 1986; Frank 1996; Skinner 1953; Bateson 1979) and mathematical isomorphisms exists between formal models of selection and formal models of learning (Harper 2009; Shalizi 2009; Frank 2009; Valiant 2013; Chastain et al. 2014).

It is common to think of learning systems as sophisticated machines (or intelligent organisms) with goal-directed intentions designed for the purpose of producing smart behaviours—which would make their abilities irrelevant to understanding evolutionary processes. But, in fact, all of the phenomena relevant to our evolutionary questions can be produced by intention-less algorithms with simple incremental improvement mechanisms. However, note that in a learning system the object of this incremental improvement process is different from a conventional optimisation process. Whereas a simple processes of optimisation (or incremental improvement) is usually applied to a solution or output directly, a learning process optimises a *model* of good solutions or outputs or an indirect representation of solutions. Evolutionarily, this is like the difference between adapting the parameters of a phenotype directly (e.g. the traits of a phenotype) vs adapting the parameters of a developmental process that produces fit phenotypes (this is a distinction which is lost when we assume a one-to-one mapping between genotype and phenotype). The learning process optimises the fit of the model to the observations (or minimises the discrepancy between the model and the observations) by incrementally adjusting the parameters of the model. The significance of this is that the model can then be used to recognise or generate new examples that have structural similarities with those that have been rewarded in the past but are not identical to them, and relatedly, examples that are far apart in ‘solution space’ can be placed next to each in ‘model space’ (Watson and Szathmáry 2015).

In advanced learning methods a model can be complicated and mechanisms for approximating the parameters of the model from observations can be quite sophisticated. But quite often the model can be simple; for example, a *correlation* model is a representation of how features in good solutions ‘go together’ or correlate. In practise, this often means making connections of some sort between different elements of a solution (causing their useage in solutions to become correlated). *Correlation learning* can be implemented via a very simple learning principle that adjusts the connections of such an organisation incrementally (Hebb 1949; Ackley et al. 1985). Mechanisms sufficient to learn a single correlation (between a pair of variables) by reinforcement can be trivial, i.e. just change a connection a small amount and see if it improves the output. This does not require sophisticated data structures, mathematical or statistical analysis, or complex computational machinery. Given variation in the connections of a network (that affects correlations between problem variables), it is simply an incremental improvement process applied to those connections. This is a crucial step-up from an incremental improvement process acting on the solution variables directly, however. Rather than merely finding good outputs, a learning process finds the structure underlying good outputs. It is this which enables new phenomenology compared to a simple optimisation process; For example, the ability to generate new patterns or behaviours that are different from the ones it was trained on but exhibiting the same underlying structure.

The consequences of correlation learning in larger systems can be surprisingly powerful. Under the right conditions, correlation learning is sufficient for the behaviours that seemed impossible for evolutionary systems (a, b and c above):

- Learning systems can perform well at novel tasks, i.e. tasks they have not previously been trained on.
- Learning systems can exhibit non-trivial collective behaviours without system-level feedback on performance.
- Learning systems can find new representations of a problem, that facilitate task learning at a higher level of organisation, before those higher levels of organisation exist.

These behaviours, described below, are uncontroversial and well-understood in learning systems, but knowledge about the conditions for and capabilities of such phenomena has not been previously transferred into evolutionary theory. Our long-term aim is to develop a unified predictive theory for these evolutionary questions by exploiting the existing concepts and extensive existing results from learning systems. It is the aim of the current paper to reinterpret the key open questions in these different biological domains from this unifying perspective, to describe how learning theory connects with these biological questions, and to review and synthesise our work thus far. In particular, we draw these works together to address their inter-related roles in major evolutionary transitions.

## Toward Unifying Principles: Connectionism and Evolution

Developmental, ecological and reproductive organisations are structures that determine which things ‘go together’ and which things are independent. Specifically, the organisation of developmental interactions governs whether it is possible for multiple coordinated changes to occur in a way that preserves their functional dependencies without causing multiple unwanted side-effects on other aspects of the phenotype. Ecological interactions specify how a change in the density of one species modifies the selective pressures acting on other species and thus govern which species are mutually exclusive and which can coexist, for example. Reproductive organisations govern whether fitness differences among the components within evolutionary units are suppressed and whether fitness differences between different evolutionary units can be inherited. Understanding the evolution of developmental, ecological and reproductive organisations thus requires that we understand how evolution alters which things *vary* together, which things are *selected* together and which things are *inherited* together, respectively.

### Connections and Correlations in Biological Networks

It is common to describe developmental and ecological interactions as networks (e.g. a gene-regulation network or food web/community matrix, respectively). These networks describe who interacts with whom, in what way and how much (i.e. how one gene-expression level affects changes in the expression of other genes, or how the density of one species affects the population growth of another species). Whilst it is not as common to describe reproductive dependencies as networks, these relationships also have the basic property of controlling how the inheritance of one evolutionary unit is, or is not, independent of the inheritance of another evolutionary unit. It is therefore useful to characterise all three (developmental, ecological and reproductive) organisations as networks. We can then ask how the structure of that network (topological changes including the strength and sign of connections, in some cases) change over evolutionary time, and how those structural changes then modify evolutionary processes.

When the structure of a network affects the dynamics that occur on it, and the dynamics that occur on the network affect changes to network structure, this is known as an *adaptive network* (Gross and Sayama 2009), e.g. where agents on a network can both choose behavioural strategies that are suitable for the current organisation, and can also choose to re-wire connections on the network to suit the current behaviours (Jackson and Watts 2002; Pacheco et al. 2006; Traulsen et al. 2008; Van Segbroeck et al. 2010). We argue that developmental, ecological and reproductive organisations (like other complex adaptive systems, Farmer 1990) exhibit this two-way property.

More specifically, we investigate the hypothesis that the evolution of developmental, ecological and reproductive organisations are all subject to the same underlying organisational principle, a simple principle of positive feedback between the topology of an interaction network and the behaviours that the network structure governs. That is, a connection between two components or nodes in this network causes them to exhibit correlated behaviour, and when nodes have correlated behaviours this causes natural selection to create or strengthen the connection between them. In short, entities that co-occur together ‘wire’ together (and entities that wire together co-occur together). For example, (as expanded below) genes that are selected together are wired together via the evolution of gene-regulatory interactions that cause them to co-vary (be co-expressed) in future, species that co-occur in high-density are wired together by the evolution of ecological relationships that cause them to be co-selected in future, and evolutionary units that reproduce together are wired together by changes to reproductive relationships that cause them to be co-inherited in future. Empirical observations are consistent with this simple positive feedback principle and in this paper we present theoretical support for each of these cases:

- Evo-devo: The more often that two genes are selected together (at the same time/in the same environment) the more selective advantage there is to strengthening developmental interactions between them. This organisational change causes their expression in the phenotype to be correlated in future (e.g. via an increase in the gene-regulatory connection between them; Wagner et al. 2007; Pavlicev et al. 2011; Watson et al. 2014; Kashtan et al. 2009). See also, for example, the principle of ‘coevolution of coexpressed traits’ and conversely ‘the rule of independent selection’ (West-Eberhard 2003).
- Evo-eco: The more often that two species populations grow to high-density together (at the same time/in the same environment) the more selective advantage there is for individual traits that strengthen ecological interactions between them. These interactions change in a way that causes their population growth to be more correlated in future, e.g. via reductions to competitive interactions between them (Lewis 2009; Power et al. 2015). For example, this type of feedback is part of the backstory involved in ‘invasional meltdown’ (Gallardo and Aldridge 2015; Simberloff and Von-Holle 1999) where species that have been in prolonged contact with one another in one environment facilitate one-another’s invasion into another environment because they “have had a long evolutionary time to develop a cosy relationship with each other” (Gallardo and Aldridge 2015). This might involve *character displacement* that reduces niche overlap when species are driven into contact (Brown and Wilson 1956; Dayan and Simberloff 2005).
- Evo-ego: The more often two evolutionary units reproduce together (at the same time/in the same environment) the more selective advantage there is to individual traits that strengthen reproductive dependencies between them. Such reproductive interactions (controlled by individual selection in either participant) cause their reproduction to be more strongly correlated or centralised in future (e.g. via evolution of co-dispersal behaviours or vertical inheritance) (Watson et al. 2009b, 2011b, submitted). For example, the ‘free-living’ ancestors of eukaryote organelles initially evolved close symbiotic relationships with the host cell (they were still separate evolutionary units at this stage, but reproducing together), and latterly became reproductively centralised and synchronised (Margulis 1981, 1993).

The consequence of this positive feedback is captured by a principle of *correlation becomes causation*. That is, variables (phenotypic characters, species populations, evolutionary units) whose behaviours originally co-varied because of a correlated external stimulus (or by accident, or because of selection acting at a lower level) come to have behaviours that co-vary because of their internal interaction structures (i.e., developmental interactions, ecological partnerships or reproductive dependencies). In the developmental domain, Riedl describes this observation as the evolution of developmental architectures that “mimic” the functional constraints on phenotypes (Riedl 1977; Wagner and Laubichler 2004). See also the conversion of alternate “ecosystem states” (configurations that are forced by changes to environmental conditions) into alternate “community states” (configurations that are intrinsic attractors of the ecological population dynamics) (Beisner et al. 2003; Power et al. 2015), and the concept of *symbiogenesis* in the evo-ego domain (Margulis 1981, 1993; Maynard Smith and Szathmáry 1995).

Although this positive feedback between topology and behaviour (organisation and evolution) is simple when considering individual connections between a pair of entities, the consequences of this principle for the dynamics of larger systems is much more interesting but not immediately obvious. However, *connectionist models* of learning and memory have characterised the capabilities and limitations of learning models based on this type of feedback in great detail. Such connectionist models can thus be used to recognise common principles across multiple domains (Farmer 1990). We show how this can be used to advance our understanding of this feedback principle in evolutionary systems—hence, *evolutionary connectionism*. Moreover, we argue that the different consequences of this feedback in evolutionary systems (affecting developmental, ecological and reproductive networks) have specific analogues in different applications of correlation learning (namely, reinforcement correlation learning, unsupervised correlation learning and deep correlation learning, respectively) that help us to understand how natural selection changes the processes of variation, selection and inheritance, respectively, in evolutionary systems.

### Connectionism in Cognitive Science

Connectionism is an approach to cognitive modelling that attempts to explain the cleverness of cognitive processes not by ascribing sophistication to the individual component parts (such as individual neurons) but to the organisation of the connections between them (Garson 2015). Artificial neural network models provide a mechanistic basis to this idea (Hinton et al. 1986; Rumelhart et al. 1986). The field of artificial neural networks has been extraordinarily successful in providing a substantially different way of conceptualising the possible machineries of memory, learning, perception and problem solving as decentralised and distributed processes (Clark 1995; Hinton et al. 1986). They have also been, and continue to be, extremely successful in providing practical machine learning methods for classification, pattern recognition, clustering, data compression and optimisation in innumerable application domains (Rumelhart et al. 1986; Hinton and Sejnowski 1999; O’Reilly and Munakata 2000; Hinton 2007), thus demonstrating that such distributed and decentralised mechanisms can exhibit computationally powerful collective behaviours.

The kind of neural networks that are relevant here are very simple and very well-studied. Such a network is characterised by a number of nodes, in a network of connections, where the activation of each node is a non-linear weighted sum of the input activations it receives from other nodes (note that in gene networks, the expression potential of a gene is generally modelled as a non-linear weighted sum of expression potentials of other genes, and in ecological networks the rate of growth of a species is often modelled as a non-linear weighted sum of other species densities; Watson et al. 2014; Power et al. 2015). The *Hopfield network*, in particular, has been used as a model for dynamical systems and emergent collective behaviours in many different domains (Hopfield 1982). This is a network where every node is potentially connected to every other node bi-directionally. Neural network models are useful to us in this context because they show that -

1. Many interesting and non-trivial collective behaviours can arise from a network of individually simple components if the connections between them are appropriately organised.
2. Organisations sufficient to produce such behaviours can arise from very simple learning mechanisms that modify connections incrementally.

These learning mechanisms gradually modify the organisation of the system by incrementally adjusting the strength of connections in the network. Modifying connection strengths in this manner has the effect of altering the correlation between the activation of one node and the activation of another. A positive connection produces positive correlation in the activation of the nodes it connects; conversely a negative connection means that when one is activated the activation of the other is suppressed. Adjusting connections in this manner is therefore a type of *correlation learning* (Hinton and Sejnowski 1999). This type of learning is just a way of implementing the very general idea of *associative learning* which has influenced cognitive modelling for centuries (Kallich 1945), i.e. learning which objects or ideas go together, or learning which stimuli go together with which outcomes, or behaviours with rewards.

In practice, neural network learning methods often calculate the appropriate change for each connection based on the observed error, i.e. the difference between desired and actual outputs—see, e.g. the Delta Rule and Back-Propogation algorithms (Rumelhart et al. 1986). Such *supervised learning* assumes that information is provided about what the correct output is during training. But this is not necessary. Correlations can also be learned via *reinforcement learning* without a priori knowledge about what the correct output is. This can be as simple as modifying connections at random and retaining modifications that improve the output. Whether it is by trial and error (reinforcement learning) or by such supervised learning calculations, the direction of change that provides improvements is the same (at least in the limit where changes affect one connection at a time). Accordingly, an equivalence with the action of natural selection can be verified (Valiant 2013; Watson et al. 2010a). In addition to improving the output of the system immediately, the consequence of such learned connections on the future behaviour of the system is to make those combinations of outputs that were rewarded in the past more likely to occur again in future (i.e. positive feedback on correlations). The subsequent behaviour of the system is thus partly determined by the current inputs at that point time, but partly determined by the past experience of the system recorded, in a distributed manner, in the network of relatively slow-changing interactions.

In contrast to supervised and reinforcement learning, some learning mechanisms are *unsupervised* which means they do not use a task-based performance metric at all (Hinton and Sejnowski 1999). One very well-known example of this employs a type of *Hebbian learning* (Hebb 1949), often paraphrased as ‘neurons that fire together wire together’—meaning that the synaptic connection between two neurons is strengthened when the neurons are both stimulated at the same time (e.g. by the same input or stimulus). Under Hebbian learning, the direction of change in the connection is determined by the current output of the system (e.g. whether the two neurons are currently firing) not by a task-specific performance metric. This type of unsupervised learning mechanism is equivalent to reinforcement learning that favours amplification (increase in magnitude) of the current outputs regardless of what they are (i.e. their signs). Because this amplification is enacted through changes to connections rather than independent variables, it has the consequence of reinforcing *combinations* of values in the current output. This causes those combinations of outputs to become more stable and resilient to perturbation. That is, if one or a small number of the system variables are changed, the weighted connections from other variables that have not changed will force it to change back (or will reduce the external input necessary to change it back). For a given distribution of initial conditions, this means that that particular combination of values is more likely to re-occur (in dynamical systems terms, the initial conditions that lead to a particular attractor state is the ‘basin of attraction’ for that pattern, and the effect of this type of learning is to increase the size of this attractor basin, i.e. to increase the number of initial conditions that lead to that attractor). Thus, whereas reinforcement learning strengthens correlations that are *good* (making changes that improve rewards and make good combinations of outputs more likely to occur in future), unsupervised learning merely strengthens correlations that are *frequent* (making changes that amplify or stabilise the current output and make those combinations of outputs more likely to occur again in future).

Hebbian learning is a fully distributed learning mechanism (i.e. the change in a connection is a function only of the activation in the two nodes it connects) and the change is not a function of system-level performance or any other system-level quantity. This type of learning nonetheless has predictable consequences for the behaviour of the network as a whole. In particular, it effectively ‘internalises’ correlations that are frequent in the input, i.e. two neurons that originally fired together because they frequently responded to the same external inputs, subsequently fire together because of the synaptic connection that has been strengthened between them. This causes the internal structure of the learning system to mimic or ‘mirror’ the structure of patterns observed in the input/external environment (i.e. learning which features of the input co-vary and which are independent), and the activation dynamics of the system to recreate patterns of activation that are ‘familiar’ given its past experience. This is called an *associative memory* (Hopfield 1982) which has many interesting properties: the ability to store and recall multiple activity patterns, to recall patterns of activation from partial stimuli, to cluster data points into intrinsically similar groups, to classify novel patterns into such categories, to repair corrupted patterns (toward the nearest training pattern), to generate generalised patterns based on structural similarity, perform dimensional reduction/data compression, and to produce idealised exemplars of class from noisy or corrupted training samples (Hopfield 1982; Hinton and Sejnowski 1999). All of these functions are consequences of the simple incremental changes to connections that reinforce frequent correlations.

Correlation learning is thus based on the same positive feedback between topology and behaviour that we observed in the other biological networks. That is, neurons whose behaviours originally co-varied because they were rewarded at the same time, or co-varied because of a common external stimulus, come to have behaviours that co-vary because of their internal interaction structures (i.e., synaptic connections). In the context of connectionist models of learning, it is clear that there are many interesting and well-understood consequences for the subsequent behaviour of the system that follow directly from this basic principle. Crucially, these consequences follow inevitably from this basic principle and are not special to neural networks; any network that exhibits this same principle at the level of individual connections, will also exhibit the same system-level behaviours. In other words, the learning algorithm that is implemented in artificial neural networks is substrate independent and will thus be instantiated in any network that has the same kind of positive feedback.

### “Evolutionary Connectionism”

We introduce the term “evolutionary connectionism” to recognise that, by processes that are functionally equivalent to connectionist models of memory and learning, natural selection acting on the relationships within and between evolutionary entities can result in organisations that produce complex system-level behaviours in evolutionary systems and improve the adaptive capabilities of natural selection over time. The basis of evolutionary connectionism is that the simple principle of positive feedback on the organisation of a system, well-understood in the context of neural network models, is also common to the evolution of developmental, ecological and reproductive organisations. This has the potential to unlock a well-established field of research, with specific conceptual and mechanistic models and results, which can be utilised to understand the evolution of biological organisations under natural selection (Farmer 1990). In particular, this framework helps us to make sense of the bi-directional feedback between evolutionary processes and structural organisations, and also to understand how the consequent changes to the organisation of the parts modifies the collective behaviour and optimisation of the whole. Hence it provides a theoretical framework where we can begin to understand how it is possible for the Darwinian machine to change its own operational mechanisms over time, and how this can improve its ability to produce adaptive change by internalising and exploiting past experience.

It is not too difficult to see that, given heritable variation in interactions and selection, evolving systems will exhibit changes to connections analogous to correlation learning by reinforcement (Watson et al. 2010a; Valiant 2013; Watson and Szathmáry 2015). The evolutionary conditions that correspond to reinforcement learning are simply those that reward changes to interactions that improve fitness (e.g. changes to a gene network that increase fitness by changing the gene expression pattern/phenotype that the network produces, Watson et al. 2014).

If the current output of the network is already the desired output of the network, or even the locally optimal output, then the distinction between reinforcement learning and unsupervised learning is moot (i.e. reinforcing the current behaviour and reinforcing the good behaviour is the same). Unsupervised correlation learning is a suitable model for evolutionary change in such cases. For example: (1) *When selection favours increased robustness.* Although there may be other quite different phenotypes that are higher in fitness to the current phenotype, *local* selective gradients may nonetheless favour changes to network structure that amplify the current phenotype or reduce phenotypic variability. If this is enacted by changing correlations (i.e. reinforcing the co-occurrence of features that already co-occur) and not merely by removing variability from individual features, this is equivalent to unsupervised correlation learning. Selection for robustness is another way of saying that the current output is the desired output, but in a *locally* optimal sense (i.e. all *small* variations are inferior). (2) *The effect of individual*-*level selection acting on interactions between different evolutionary units within collectives* (e.g. individuals within an ecological community). Because individuals adopt behaviours that (locally) maximise individual fitness, individual selection favours variations that enable them to retain their current behaviour (or do more of the same behaviour). This is not, in general, good for the fitness of the collective (the sum of individual fitnesses); whenever individual behaviours are subject to a social dilemma, the behaviours adopted under individual selection do not maximise collective fitness. Nonetheless, in reinforcing the current behaviour of each individual the configuration of the collective is made more stable or robust. If these changes are enacted by altering the coordination of social behaviours with other individuals, and not merely by altering individual behaviours, this is equivalent to unsupervised correlation learning at the system level; i.e. reinforcement learning at the individual level can produce unsupervised learning at the system level (Power et al. 2015; Watson et al. 2010a). This is yet another way of saying that the current behaviour is the desired behaviour—but here the point is that the current behaviour of the system is the locally desirable behaviour for *individuals* even if it is not ‘the desirable behaviour’ for *collective* fitness. In sum, both reinforcement correlation learning and unsupervised correlation learning mechanisms can occur in evolutionary systems; unsupervised correlation learning results from selection for robustness at the system level or from selection acting at a lower level (maximising the utility of the components rather than the collective).

It is not a coincidence that learning systems and evolving systems exhibit the same organisation principles. The reason that connections are changed by reinforcement (or unsupervised) learning in the direction that they are is because that is the direction that improves the output of the system (or amplifies the current output). And the reason that natural selection evolves changes to connections in the same direction is because that is likewise the direction that improves the output of the system (or retains its current fitness levels). It is simply the result of selection for good or non-worse patterns of correlation. By adopting structural configurations that mimic the selective environments they have experienced, or by canalising their current response to it, they can increase fitness or prevent it from being decreased. The consequence of these selected changes is that evolving systems internalise information about the environment (e.g. what combinations of phenotypic features are fit, or what combinations of species can coexist given the prevailing abiotic conditions).

In fact, this kind of change is so basic and natural that it occurs spontaneously in any dynamical system built from a network of malleable interactions—in this sense, neural and evolutionary organisations are both examples of the same underlying principles. For example, even a purely physical system of particles and springs, where the springs, as well as being elastic, are also slightly plastic (e.g. weaken with stress), will exhibit this type of positive feedback (we assume that the spring constants change slowly compared to changes in the particle states that they control). This does not require natural selection; rather, spring-changes merely minimise energy (i.e. adopt a configuration that causes them to do less work, a.k.a. wearing out or deforming under forcing) given the structure of the perturbations exerted on them by the environment. We argue that organisations evolving under natural selection necessarily follow the same basic principle. Each adopts an organisation that reflects the structure of the environment that they are exposed to because organisations that do not will be changed by the action of the environment (Ashby 1956, 1960; Godfrey-Smith 1994; Gell-Mann 1994).

Whereas the consequences of this type of feedback in developmental, ecological and reproductive organisations is very poorly understood, the consequence of this type of feedback in neural networks has been thoroughly characterised by decades of research. This enables us to understand how the action of this feedback on individual connections modifies the organisation and dynamical behaviour of the system as a whole—in particular, its problem solving or optimisation capabilities.

### Learning How to Adapt

A learning system improves its performance at some behaviour (e.g. classification, clustering, foraging) by incrementally optimising the parameters of that behaviour. Although the behaviour being improved is not usually an optimisation process, it can be. Our recent work shows how a learning system can learn how to optimise more efficiently and effectively over time by beneficially biasing the parameters of the optimisation process (Watson et al. 2010b, 2011a, b). For an optimisation process based on trial and error (formally, ‘generate’ and ‘test’, Watson et al. 2011b), there are logically two different ways in which the trajectory of the search process through solution space can be biased: (a) by changing the test function or selection function, i.e. the way new solutions are evaluated or their apparent value, or (b) by changing the way new solutions are generated. Intuitively, changing the apparent value of a solution (e.g. by artificially increasing the value of certain combinations of variables that are ‘familiar’ given past experience), may enable an optimisation process to escape a local peak in a reward function by raising the value of some points and/or lowering others. Whereas, changing the way solutions are generated (e.g. by re-using a particular combination of variables, i.e. a module, and substituting it for another) may enable an optimisation process to escape a local peak in a reward function by creating directed ‘jumps’ in solution space (Watson et al. 2011b).

#### Learning How to Adapt by Changing the Selection Function

This approach can be demonstrated by combining two previously unrelated behaviours, each independently well-understood in the Hopfield network but not previously brought together (Watson et al. 2009a, 2010b):

(i) The connections of a (non-learning) network can be defined to represent the constraints of a constraint optimisation problem, and running the network with this interaction structure causes it to find activation patterns that are locally optimal solutions to that problem (Hopfield and Tank 1985, 1986).
(ii) A learning network shows an ability to form a generalised memory of past experience by internalising correlation structures observed/experienced in that environment (Hopfield 1982, Fontanari 1990).

Combining these two behaviours in the same network (but on different timescales) defines an interesting new kind of dynamical system. This combines fast state dynamics (with occasional perturbations) and relatively slow changes to connections. Initially the behaviour of the activation dynamics merely finds locally optimal solutions to the problem, as in (i). But at the same time the network is learning. It is not learning a predefined set of patterns as in (ii), however. Rather it is mimicking the patterns of activation that are found at locally optimal configurations. We call this a *self*-*modelling system* (Watson et al. 2010b) because the changes to its connections effectively form an associative memory of its own behaviour. This bidirectional feedback between the behaviour of the system and the organisation of the system means that as the organisation of the network begins to change, it changes how the system behaves, and hence changes the solutions that it finds. Specifically, we show that this causes the system to find better solutions more reliably over time, and can enable it to find high-quality solutions that would otherwise be, not just found less frequently, but highly unlikely to be found at all (Watson et al. 2010b). In some cases, unsupervised correlation learning (or equivalently, reinforcement learning applied at the component level), is sufficient to attain these behaviours.

The explanation of why a self-modelling system finds better solutions over time has three parts (Watson et al. 2009b, 2010b, 2011a). First, in systems involving many low-order (e.g. pairwise) constraints, the size of dynamical attractors (if there are multiple attractors) is positively correlated with their depth (i.e. low energy attractor states can be reached from more initial conditions than higher energy attractor states). For this not to be the case the slope of the energy landscape would have to be arbitrarily steep, and this cannot be the case when the energy function is built from the sum of many low-order, e.g. pairwise, interaction terms each of limited magnitude. Second, the positive feedback between the activation dynamics on the network and changes to the topology of the network means that configurations that are most frequently observed are most frequently reinforced. Given the first point, this increases the likelihood that the system finds good configurations that it has found in the past, and decreases the likelihood that it will find inferior solutions over time. Third, and more interestingly, correlation learning forms a *generalised* associative memory of a set of patterns (Fontanari 1990) and not just a ‘rote’ memory. This means that if there is an underlying structural regularity common to easy-to-find locally optimal solutions that can be represented in a correlation model, then the structure that the network learns will reflect the correlation structure of those patterns and not the patterns *per se*. The network is therefore able to favour novel patterns with this underlying regularity and is not merely limited to favouring the specific patterns it has already discovered. Thus, by mimicking the correlation patterns observed in the problem the system can generalise over a distribution of easy-to-find locally optimal solutions to enlarge the basin of attraction for exceptionally high-quality solutions even before such solutions have been visited for the first time (Watson et al. 2010b). In terms of adaptive systems more generally, these conditions create a link between simple habituation behaviours (that canalise the current system state or make it more robust) and adaptation that generates and exploits novel configurations that may be superior to any previously experienced past state. In evolutionary terms, this is the link between robustness and evolvability.

Computationally, this is a simple form of model-building optimisation (Pelikan et al. 1999; Hauschild and Pelikan 2011). These are techniques that learn a model of the problem structure and then use this structure to find better solutions to the problem. But in this case, this occurs without using specialised machine learning mechanisms. It uses only very simple positive feedback principles, based on incremental changes to connections that are generic across a broad range of adaptive networks (Watson et al. 2010b, 2011a; Mills 2010).

#### Learning How to Adapt by Changing the Generate Function (‘Deep Optimisation’)

Although the optimisation capabilities of self-modelling dynamical systems are demonstrably superior to those of non-learning networks, the capabilities of non-hierarchical systems are limited. Specifically, if a problem has structural regularities that cannot be represented in pairwise correlations, incremental modification of pairwise connections will not be able to capture or exploit this regularity. However, deep or multi-layered networks (Hinton 2007; Rumelhart et al. 1986) can represent higher-order structural regularities that cannot be represented in single-level networks.

The problem-solving principles underlying deep learning are related to problem decomposition and ‘chunking’ (Mills 2010; Mills et al. 2014; Watson 2006), i.e. breaking a complex or high-dimensional problem down into more manageable sub-problems and then assembling together the solutions to these sub-problems in different ways to solve larger problems, and so on. The tricky thing is how to do this ‘bottom-up’ i.e. without a teacher, providing knowledge of how to decompose the problem, to guide the learner through the necessary steps. In neural networks, this type of hierarchical learning is recently referred to as ‘deep learning’ (Hinton 2007; Hinton et al. 2006; Hinton and Salakhutdinov 2006), but the ambition to build deep learning models has been around a long time, e.g. by building networks with many layers; each layer taking inputs from the previous layer, transforming it into a new representation, and passing that on the next layer (Rumelhart et al. 1986). In these cases the overall learning task is often supervised (i.e. a performance metric on the output layer is used). However, the trouble with conventional approaches is that assessing the error on the output of the network massively underdetermines the changes that are required on intermediate layers (a.k.a. hidden layers) of the network (Rumelhart et al. 1986; Hinton 2007). That is, there are many different possible intermediate representations that can give the same outputs for a given input (but only some of these will generalise well). Moreover, it is not clear how to devise an appropriate supervised learning function that operates directly on the intermediate layers (that is, an intermediate representation that is ‘correct’ for one output layer may be useless for a different output layer). An exciting new development in neural network research, *Deep belief networks* (Hinton et al. 2006; Hinton 2007), has revived interest in deep learning by providing a new approach to this problem. This technique uses *unsupervised* learning to build intermediate levels of representation one at a time, and ‘freezes’ what has been learned in each layer of connections before the next level of organisation is added on top. This exploits the ability of unsupervised learning to find representations that mimic the intrinsic structure of the problem (without feedback on performance) to reduce the dimensionality of the problem before higher-level layers are constructed. This low-dimensional representation can make task-learning at the next level easier, and thus provides good generalisation for supervised learning at a higher-level of the network even though that level of the network didn’t exist at the time the lower layer was learned. In this manner, a combination of unsupervised and supervised learning is far more computationally efficient than either alone.

Using these deep learning ideas to improve an optimisation process leads to what we might term *deep optimisation* (closely related to *multi*-*scale search*, Mills et al. 2014). The idea here is a process that first uses a simple hill-climbing optimisation process to find local optima, then learns and exploits low-order regularities over a distribution of these optima to find better optima, then learns higher-level regularities from a distribution of these optima to improve optimisation further, and so on. We have demonstrated that this type of learning can efficiently solve optimisation problems that are provably difficult for single-scale search (Mills 2010; Mills et al. 2014). This technique exploits the multi-scale aspect of an individual-based evo-ego model (Watson et al. 2009b, Watson et al., submitted) and implements it in a machine learning method (without the need for an individual-based simulation model). Related but more sophisticated principles are involved in grammar-based optimisation methods. Here a *schema grammar*, representing the hierarchical correlations observed in a distribution of above-average solutions, is learned and exploited to re-scale the search process at a higher level of organisation (Cox 2012; Cox and Watson 2014).

A deep optimisation approach can also be implemented in a fully distributed neural network model using only local learning mechanisms. This is an extension of the self-modelling dynamical system above and learns connections in exactly the same way, i.e. by reinforcing connections that are frequently observed at local optima. The difference is in what the connections mean—their causal role in the dynamics of the system behaviour. In the simpler self-modelling system, changes to connections have the effect of altering the fitness function of the optimiser, or equivalently, the energy function of the dynamical system—making familiar configurations lower energy (i.e. more likely to be retained). In the ‘deep’ version of the self-modelling dynamical system (a.k.a. “rHN-g”, Watson et al. 2011b), changes to connections do not alter the energy function but they alter the way movements in configuration space are sampled. Specifically, the learned links are used to create clusters of state variables that vary in a coordinated fashion. These higher-level units, or modules, change subsets of variables simultaneously, and these multiple state changes will either all be kept or all rejected, as a unit, depending on the change in energy/fitness that they confer. This enables directed jumps in configuration space—changes to many variables that may be beneficial in combination even if each individual variable-change involved is individually deleterious. Higher-level, clusters of clusters can be created recursively or hierarchically in the same way. This has been shown to solve modular constraint problems that cannot be solved by self-modelling networks that do not have the ability to do this recursive encapsulation (Mills 2010; Mills et al. 2014; Watson et al. 2011b). These optimisation techniques provide a solution to the problem of inventing a representation that makes a problem easy to solve by *recoding* the original high-dimensional problem into a lower-dimensional representation of the problem space (Watson et al. 2011b). The positive feedback principle is still apparent in the way connections are learned and used; i.e. variables whose values are frequently correlated become ‘wired’ together in such a way that, after they have been connected, they cannot vary independently—they are transformed into a new emergent or higher-level state variable.

Importantly, the reason that a new connection is made is not, and cannot be, because this connection will, at some point in the future, provide jumps in configuration space that are adaptive; a benefit that has not yet happened cannot be the reason the connection was made. The connections that are made simply canalise the co-occurrence of variables that already co-occur (and are therefore close to neutral at the time they are made). Over a distribution of local optima, the connections that are most robust (most often neutral) are retained by the learning algorithm and those that are less robust (occasionally deleterious) are removed. This deep optimisation technique thus exploits the unsupervised correlation learning principle of deep learning: connections that canalise existing correlations reduce the dimensionality of the problem space in a way that is effective in enabling adaptive jumps in configuration space (or movements in a higher-level representation of the problem), even though those higher-level jumps have not yet occurred.

Together these works show that learning can improve optimisation. This can be achieved by using learning to bias selection, i.e. altering the effective energy function (or the fitness function) that controls the dynamics of the system. Or it can be achieved by using learning to bias the movements that are sampled in configuration space, e.g. by exploiting modularity or, in particular, by collapsing subsets of variables into higher-level emergent variables (Watson et al. 2011b). The deep optimisation provided by changing the generate function has optimisation capabilities that cannot be exhibited by changing the selection function. Next we argue that these different types of connectionist learning are implemented in the evolution of different types of biological organisations, and that they have analogous consequences for the adaptive capabilities of evolution by natural selection.

### Evolutionary Connectionism and the Level of Evolutionary Unit

We argue that specific but different types of correlation learning are directly relevant to understanding the evolution of developmental and ecological organisations, and to understanding how they work together with the evolution of reproductive organisations in the evolutionary transitions.

The evolution of developmental and ecological organisations are very different from each other because of the level of organisation that constitutes the evolutionary unit in these two cases. Developmental networks, controlling the combinations of features that co-occur in phenotypes, are selected as a single evolutionary unit; selection applies at the level of the whole network and changes in the organisation of the network can be selected because of the differences they confer on the phenotypes that are produced. This is the analogue of reinforcement correlation learning. In contrast, ecological networks are not evolutionary units, and when we talk about the evolution of the ecological network we just mean the changes, caused by evolution, that occur as a consequence of natural selection acting on the composition of each species within the ecological community individually not as a composite unit (in short, no group selection here). Because selection occurs at the individual level and not the system level, this is the analogue of unsupervised correlation learning—selection does not act to increase collective fitness (in general) but in acting to increase individual fitness (by changing community interaction terms) it can have the (side-)effect of stabilising the current ecological composition or increasing ecological robustness. Interestingly, the observation that selection for robustness at the system level also follows unsupervised learning principles seems to imply an underlying equivalence between selection for robustness at the system level and selection that acts on components below the system level—e.g. selection that favours modularity or features that are independently beneficial is closely related to selection that favours robustness at the system level. But importantly, system robustness produced by changes to interactions does not simply remove variability, but shapes or directs variability at the system level—in the same way that unsupervised correlation learning mimics correlations in the input.

Our work described below suggests that the evolution of developmental and ecological interactions are both analogous to self-modelling systems that alter the selection function—but whereas changing ecological interactions explicitly alters selection on evolutionary units by altering the relationships they have with other units (ecological relationships between species in the community), changing developmental interactions alters ‘selection’ on components that are internal to the evolutionary unit (e.g. on gene-expression potentials within the organism). The latter has the effect of biasing the phenotypic variation that is produced at the system level, i.e. by changing the co-occurrence of traits in the phenotype.

The evolution of reproductive relationships is not captured by any one level of evolutionary unit—neither the system as a whole nor the individual components. Rather, the evolution of reproductive relationships is precisely concerned with changes in the evolutionary unit—changes that convert multiple evolutionary units at one level of organisation into a new evolutionary unit at a higher level of organisation. These changes must be driven by the lower level evolutionary unit—the higher level unit cannot be driving the evolutionary process before it exists (Ryan et al. 2015). We argue that this is analogous to the incremental addition of new layers of organisation in deep learning (combining unsupervised and supervised correlation learning mechanisms applied at successive scales of organisation) or to the rescaling of the optimisation process as per the distributed deep optimisation models. This biases the combinations of particles that are created in a more radical (multi-scale) manner than the evolution of developmental interactions (Watson et al. 2011b). Specifically, whereas developmental organisations bias *phenotypic* variability (by recreating specific phenotypic patterns through the organisation of internal selection or context-sensitive differential growth between components) (Laland et al. 2014), reproductive organisations can bias *genetic* variability (by enabling the combination of genetic differences in a collective to be inherited to descendent collectives as a unit and supressing internal differential selection between them).

Accordingly, although evo-devo, evo-eco and evo-ego domains each have their own special characteristics, there are methodological reasons to study them as a set:

1. They have common underlying principles that can be captured by adaptive networks. In particular, a positive feedback between topology and behaviour (i.e. correlated behaviours result in the evolution of stronger network interactions which in turn produce more strongly correlated behaviours, and so on).
2. Learning theory offers detailed knowledge about the consequences of this type of positive feedback in networks, namely correlation learning—offering unifying principles for studying the evolution of these three classes of biological networks.
3. Learning theory also offers well-studied variants of correlation learning (reinforcement correlation learning, unsupervised correlation learning and deep correlation learning) that apply to developmental, ecological and reproductive organisations, respectively—offering principles that help us understand the algorithmic distinctions between the three cases.

Studying them as a set is also motivated by their complementary roles in the major evolutionary transitions:

(4) Taken together, developmental, ecological and reproductive organisations cover the evolution of the necessary and sufficient components of the Darwinian machine, namely, variation, selection and inheritance, respectively.
(5) The three organisations are complementary in the level of evolutionary unit they address: evolution of a network as a single evolutionary unit (evo-devo), evolution of multiple evolutionary units within a network (evo-eco), and evolution that changes the evolutionary units (subsets of nodes become single nodes) (evo-ego).
(6) Together they control how information about the selective environment (e.g. what things ‘go together’) is broken-up into smaller pieces (evolution of developmental modularity or ecological independence/community structure) and put together into larger systems (evolution of developmental and ecological dependencies), and ultimately re-scaled to enable evolutionary adaptation to be reinstantiated at a higher level of biological organisation (via evolutionary transitions).

A summary of the unifying themes and their relation to principles of connectionist learning is given in Table 1.

**Table 1:**
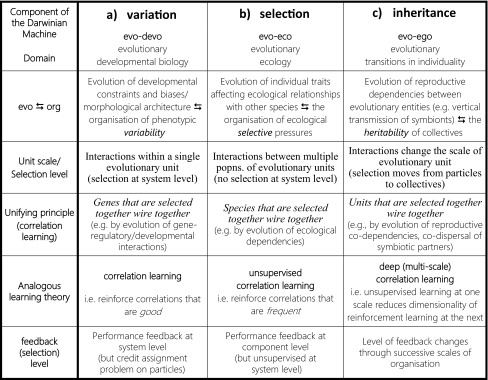
Outline of a connectionist theoretical framework for the evolution of developmental, ecological and reproductive organisations

The simple principle of positive feedback between behaviour on a network and changes in network topology, known as correlation learning in neural networks, aka. “Neurons that fire together wire together”, is analogous to the evolution of developmental, ecological and reproductive organisations

In the following sections we briefly discuss how our work using this framework is beginning to answer the motivating questions in evo-devo and evo-eco. We then discuss our preliminary work on the evolution of new evolutionary units and major transitions in more detail.

## Developmental Organisation and Evolvability

Rupert Riedl, one of the founding pioneers of the field we now know as evo-devo, suggested that body plans and developmental constraints evolve to mimic the structure of the constraints imposed on the phenotype by the environment (Riedl 1977; Wagner and Laubichler 2004). Only recently has theoretical work caught-up with this intuitive idea. Toussaint and von Seelen (2007) show that natural selection necessarily favours phenotypic variation distributions that are structurally similar to the pattern of selection. Pavlicev et al. (2011) analyse the population genetics of selection on an allele controlling the correlation of two phenotypic traits. They show that it evolves under natural selection to align phenotypic variation with the direction of selection, such that if two traits are selected together positive developmental correlations evolve, and if one is selected for when the other is selected against negative developmental correlations evolve. This is functionally equivalent to reinforcement correlation learning. In larger systems we see the same pattern occurring in work where developmental interactions evolve modularity that to mimics the modular structure of variation in a selective environment (Lipson et al. 2002; Watson et al. 2014; Kashtan et al. 2009).

Building on these observations, our work shows that the regulatory interactions of gene-regulation networks evolve under natural selection in exactly the same manner as correlation learning modifies the synaptic connections of a neural network (Watson et al. 2014). This enables us to show that gene-regulation networks can exhibit a “developmental memory” able to store and recall multiple phenotypes that have been selected for in the past, exactly like the associative memory of a Hopfield network. Using knowledge of how correlation learning can generalise over a set of training patterns, we also showed that gene-networks can produce novel phenotypes that have not been selected for in the past but have common structural regularities (e.g. new combinations of phenotypic modules). This provides a more formal basis with which to understand Parter et al.’s results (2008) showing that genotype-phenotype maps can generalise from past environments to facilitate evolvability in novel environments.

The analogy between generalisation in learning systems and evolvability in novel environments has considerable technical depth. For example, generalisation in learning systems is not mysterious, and accordingly, the evolution of evolvability that facilitates long-term adaptation in previously unseen environments is possible (Watson et al. 2010a; Kounios et al., in prep.). But neither is generalisation for granted in learning systems, and learning theory can help us understand the capabilities and limitations of such generalisation and evolvability in evolved systems. In particular, in learning systems, *overfitting* occurs when improved performance on the training set decreases generalisation on the test set. This is the analogue of past selection that fails to facilitate future evolvability. However, conditions that alleviate overfitting and improve generalisation in machine learning, such as the application of a parsimony pressure that favours simple models, also confer better generalisation in evolved G-P maps (e.g. applied via a cost of connections; Clune et al. 2013) (Kouvaris et al. 2015).

## Organisation in Ecological Communities Without Selection at the Community Level

Evolutionary processes exhibit different outcomes in structured populations than they do in freely-mixed populations. Often, this is modelled with games played on networks (Jackson and Zenou 2014). Recently there has been a rapid expansion of work investigating what happens when agents on a network have behaviours that can alter the topology of the network (Pacheco et al. 2006; Traulsen et al. 2008; Van Segbroeck et al. 2010; Santos et al. 2006), i.e. *adaptive networks* (Gross and Sayama 2009). Different scenarios involve different assumptions about exactly how agents modify their connections to others, but most cases are consistent with the assumption that they change connections to maximise their individual utility (e.g., leaving connections unchanged when they are winning, and re-wiring connections when they are losing; Nowak and Sigmund 1993; Santos et al. 2006).

Usually these models are intended to represent the interaction structure of a single population where nodes represent individuals and edges represent fitness-affecting interactions between individuals. If instead, we utilise a network model to represent ecological interactions, then nodes represent different species and edges represent ecological fitness interactions between species. When the network represents a single population, the reproduction of an individual at one node may replace the individual of a connected node (because they are members of the same species). But in an ecological network this is not the case; reproduction of individuals simply modifies the strategy of the species at that node (and/or the connections the species has with others). We have shown that a games-on-networks model of this type behaves exactly like unsupervised correlation learning (Davies et al. 2011; Watson et al. 2010a). Moreover, since the total community welfare of a social attractor is correlated with the basin size of the attractor, the changes to connections produced under an individual utility-maximisation principle have the emergent effect of increasing global community welfare (collective fitness) without selection at the system level (Davies et al. 2011; Watson et al. 2010a)—as per a self-modelling dynamical system.

We can also model the evolution of ecological organisations in a more conventional Lotka-Volterra system where a community matrix represents the network of ecological interactions (Poderoso and Fontanari 2007; Wilson 1992). We then let individuals within each species carry traits that can modify their ecological interactions with others under individual selection (i.e., in whatever manner increases their representation within their own species). This work shows that interactions evolve according to the principles of unsupervised correlation learning. This is consistent with character displacement (Brown and Wilson 1956; Dayan and Simberloff 2005) which alleviates competitive interactions or reinforces mutualistic interactions in proportion to the contact a pair of species has experienced. Accordingly, an ecosystem can form a distributed associative memory of past environmental conditions, storing and recalling multiple community composition patterns in the resultant population dynamics, despite the fact that there is no selection for such behaviour at the system level (Power et al. 2015). This suggests a significant expansion for the role of ecological memory observed in natural ecosystems (Thompson et al. 2001) and provides the possibility of being able to characterise the conditions under which eco-evo dynamics lead to self-regulation/homeostasis rather than self-destruction (Lenton 2004; Lenton and van Oijen 2002).

## The evolution of New Evolutionary Units

Conventionally, it is assumed that the products of the Darwinian machine do not alter how the machine operates, i.e. that fixed mechanisms of variation and selection are applied to a fixed reproductive unit. Above we have discussed how changes to developmental organisation and ecological organisation can alter variation and selection. But in an evolutionary transition, all three of these component mechanisms (i.e., variation, selection and inheritance) are transformed or re-created at a higher level of organisation. This results in a Darwinian machine that operates via heritable variation in reproductive success at a higher level of biological organisation. There are many complex issues involved, and the details are different in different types of transition. Nonetheless, we discuss how the basic positive feedback principle applies in this context, i.e. evolutionary units that reproduce together (at the same time or under the same conditions) become ‘wired’ together into a new evolutionary unit, and how connectionist learning principles help us understand the consequence of this feedback in larger systems.

First, we discuss the evolutionary challenges involved in different types of transition and, in particular, collectives containing particles of complementary functional types. We explain why coordinating these complementary functional types is fundamental to creating fitness differences that belong to the collective. Second, we discuss our work thus far in the evolution of pairwise relationships, and third, our preliminary work on the evolution of new reproductive units in larger networks.

### The Evolutionary Challenges in Egalitarian and Fraternal Transitions

#### Types of Transitions

Evolutionary transitions can be classified into two types (Queller 1997):

- *Fraternal transitions*, e.g., the transition to multi-cellularity, involve controlled aggregations of related individuals (‘like kinds’). Their origination is motivated primarily by economies of scale. This involves the evolution of traits that modify relatedness by evolving parameters of population structure that control the amount of mixing (such as initial group size/life cycle bottleneck size, germline segregation, dispersal parameters/time in groups) (Jackson and Watson 2013; Powers et al. 2011; Ryan et al. 2015; Johnson and Gaines 1990) and hence the likelihood of meeting others like oneself (assortment).
- *Egalitarian transitions*, e.g., the symbiotic origin of eukaryote organelles (Margulis and Fester 1991; Margulis 1993) and the origin of chromosomes (Maynard Smith and Szathmáry 1993), involve the union of previously unrelated lineages (‘unlike kinds’). Their origination is motivated primarily by the provision of complementary functions (Bouchard and Huneman 2013). This involves the evolution of reproductive mechanisms that change the co-dispersal of lineages (e.g. changing from horizontal to vertical transmission of symbionts), linking lineages that were previously inherited independently such that they are subsequently inherited together.

**Fig. 1:**
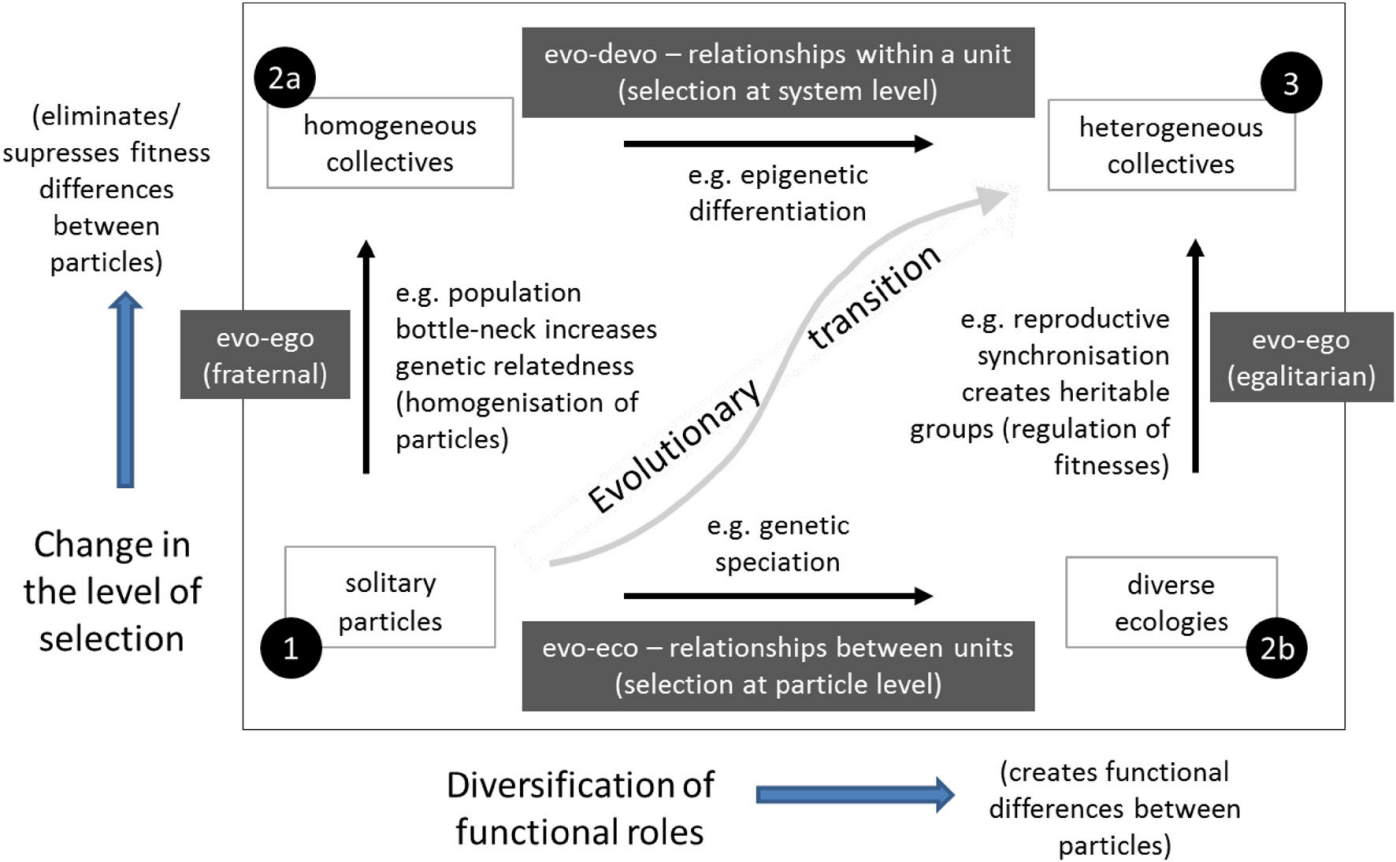
Two dimensions of change in evolutionary transitions. Fraternal transitions (e.g. to multicellularity) involve first a change in the level of selection, then diversification of functional roles within this new unit (1 → 2a → 3). Egalitarian transitions (e.g. to eukaryote cells with organelles) involve first a diversification of functional roles between multiple units then a change in the level of selection (1 → 2b → 3). Whereas conventional views of the transitions focus on changes in the level of selection (*vertical axis*), this view emphasises how the evolution of developmental and ecological relationships (*horizontal axis*) creates organisations that govern the complex phenotype of the new unit/collective. This coordination of diverse functional roles between particles within a collective is essential to create fitness differences between collectives whilst simultaneously eliminating or suppressing fitness differences between particles

These differences suggest that these types of transition do not share common mechanisms or motives (Queller 1997). However, both types of transition, when considered more fully, involve both a change in the level of the evolutionary unit (from particles to collectives) and the origination of heterogeneous functional roles, but in different orders (Fig. 1). In egalitarian transitions, evolutionary entities differentiate functional roles first (e.g. via speciation) and then form a new evolutionary unit, whereas in fraternal transitions, entities change the scale of the evolutionary unit first and then differentiate in their functional roles. Both types of transition thereby result in higher-level groups with internally differentiated roles, and organisational changes controlling the diversification of roles and changes in the level of selection are involved in both types of transition. In egalitarian transitions this diversification of roles and the coexistence of complementary types (occurring before the transition) involves changes in relationships that are *ecological*—i.e. between multiple existing evolutionary units. In fraternal transitions the diversification of roles (occurring after the transition in the evolutionary unit) involves changes in relationships that are *developmental*—i.e. within a single unit of selection. The evolution of organisational networks is central in both cases, however. An important difference between fraternal and egalitarian transitions is that in the differentiate-first egalitarian case the differentiation of roles can be controlled by the original inheritance mechanism (e.g. mitochondria are different from the nucleus because they have their own genetic lineages), whereas in the differentiate-second fraternal case, the differentiation of roles must be controlled by a new inheritance system (e.g. liver cells are different from skin cells because of their epigenetic states) (Jablonka 1994; Jablonka and Lamb 2006).

The observation that all transitions involve both changes in the level of evolutionary unit and diversification of roles is logical given what is required to instantiate the Darwinian machine at a higher-level of organisation, in particular, the suppression of fitness differences at one level of organisation and the creation of heritable variability that confers fitness differences at another (Clarke 2010, Ryan, in prep.). Much work on the evolution of individuality focusses on factors that suppress fitness differences between particles within a collective in order to prevent particle competition within groups from subverting group heritability (Okasha 2006; Bourke 2011). For example, the origin of multicellularity (in most cases) involves the evolution of a population bottleneck and sequestration of the germ line that creates high-relatedness among particles within a collective and facilitates high frequencies of cooperative particles (Godfrey-Smith 2009; Buss 1987; Okasha 2006). But this is only one aspect of a transition. It is also necessary that these cooperative particles have the capability to take on heterogeneous roles in the group phenotype (Jablonka 1994; Jablonka and Lamb 2006), enabling fitness advantages to arise from the coordination of those roles (e.g. via division of labour). In multicellular organisms this is facilitated by epigenetic differentiation that enables cells to adopt heterogeneous phenotypes despite being genetically homogeneous (Jablonka 1994; Jablonka and Lamb 2006). This separation of genotype from phenotype (i.e. requiring a plastic developmental process) is necessary to create a situation where fitness variance within collectives can be suppressed without also eliminating fitness variance between collectives (Tudge et al. 2013; Jablonka and Lamb 2006). That is, for there to be selection at a higher level there must be fitness differences between collectives, yet collectives must contain particles that all have the same replication rate (to prevent individual selection from undermining the collective). If collectives only contain one type of particle (i.e. fraternal), then this removes within-collective variation but also makes it more difficult after the transition for descendent collectives to be different from each other (unless particles have coordinated plastic phenotypes). Conversely, if collectives contain more than one type of particle (i.e. egalitarian), then descendent collectives can be different because of the particular combinations or arrangement of particles they contain but selection on within-collective variation must be controlled (e.g. by ‘policing’ mitochondrial replication).

#### Fitness Differences that Belong to the Collective

In order to identify natural selection at a new level of organisation we want to distinguish it from changes that are driven by natural selection at the existing (lower) level of organisation (Okasha 2006). Only if collective interests and particle interests act in opposition to one another could an adaptation be observed at the collective level that could not be explained as the result of adaptation at the particle level. However, at the same time, it seems counter intuitive to assert that particles belonging to fit collectives must be individually unfit. Indeed, we might expect that the fitness of a particle might be directly derived by its membership in a fit collective—implying that particle fitness is proportional to collective fitness, and collective fitness is a linear sum of particle fitness. There are conflicting ideas here that need to be disentangled. Let us first make the issues more concrete with an example.

Consider an example where particles attain fitness benefits by having phenotypes that are *coordinated* with the phenotypes of others (complementary roles), rather than benefits that arise from their intrinsic individual characteristics. For example, suppose that a proto-multicellular organism must be both motile (in order to gather resources to survive) and fecund, and that individual cells cannot be in the motile state and in the reproductive state simultaneously (Solari et al. 2006). Both roles might be provided initially in a single-celled organism via phenotypic plasticity and a lifecycle that moves reversibly between one state and the other. But if two cells work together to allow specialisation in these roles, there are efficiencies to be gained in, for example, the time and energy required to switch between phenotypic states. Clearly, the fitness of an immotile reproductive cell or a non-reproductive motile cell alone may be zero. But the fitness of cells that belong to a collective (of two) with complementary roles is non-zero (for the non-reproductive cell, it is its inclusive fitness that is relevant; Ryan, in prep.). Accordingly, their individual fitness in this case is not depressed in order to serve the fitness of the collective; quite the opposite, they are fit because they belong to a fit collective (Ryan, in prep.). Yet, if collective and individual fitnesses are in alignment, this seems to undermine the significance of adaptation at the collective level (Okasha 2006).

Okasha (2006) points to the resolution. Specifically, even if collective fitness is a linear sum of particle *fitnesses*, collective fitness can nonetheless be a non-linear function of particle *phenotypes*. In the previous example, the reproductive cell state is a phenotype that has a fitness that can be very high or very low depending on whether the other cell in the partnership adopts the motile cell state or reproductive cell state, respectively. The cell *phenotypes* thus have a highly non-linear relationship to collective fitness, but individual *fitness* (fitness of the particle, not fitness of the particle-phenotype) may nonetheless be proportional to collective fitness. Thus when particles have diverse, potentially synergistic, functional roles, good coordination between them can create fitness benefits at the collective level that cannot be accounted for by phenotypes that confer fitness differences at the individual level. It is thus the *coordination ability* itself that is both fit for the particle and fit for the collective, whereas neither of the two cell-phenotypes are individually fit (more exactly, fitness differences between these particle phenotypes do not explain the fitness differences that can arise between collectives). Accordingly, the ability to coordinate particle phenotypes with one another is not just a useful ‘add-on’ in evolutionary transitions, but actually essential in creating fitness differences that belong to the collective and not to the lower level of biological organisation. Accordingly, the evolution of individual particle phenotypes is inadequate to explain collective-level adaptations, and it is in exactly this case where a connectionist approach, i.e. addressing the evolution of relationships that coordinate particle phenotypes, comes into its own. In egalitarian transitions this requires coordinating the reproduction and inheritance of diverse types within collectives. In fraternal transitions this requires particles that are plastic; capable of producing more than one phenotype, i.e. a context-sensitive developmental process, possibly including parental effects (Tudge et al., submitted, Tudge et al. 2013).

#### Minimal Formal Models of ‘Coordinated Functional Roles’

Collective fitness thus requires the presence of more than one mutually-exclusive particle phenotypes that are complementary or synergistic (e.g. specialisation in survivability and fecundity). Let us denote these particle phenotypes as 0 and 1, such that collective phenotypes may be 00, 01, 10, or 11 (01 and 10 may have the same fitness). For each of these particles, neither the 0 phenotype nor the 1 phenotype is successful on its own, nor is it any more fit than the other phenotype on average over the possible contexts provided by the other particle. Nonetheless, by evolving to coordinate particle phenotypes (or anti-coordinate them) a collective containing complementary particles may be fitter than other collectives. This will thereby confer a fitness benefit to the particles it contains even though these fitness benefits cannot be attributed to either particle phenotype. In machine learning, a function with this property is called a *non*-*linearly separable* function—and is a well-known touchstone in neural learning test cases. The non-linearly separable functions for two Boolean variables are logical XOR (inequality, 01 or 10) and IFF (equality, 00 and 11). Other functions do not have this property. For example, under logical AND (11), a 0 phenotype is never superior to a 1 phenotype, but a 1 phenotype is superior to a 0 phenotype in some contexts (i.e. contexts where the other particle is a 1); accordingly, particle-level selection alone can produce the 11 collective.

Interestingly, when groups are built of individuals from two different species (i.e. egalitarian transitions), XOR and IFF are logically isomorphic because the labelling of each variable (0 or 1) is arbitrary and independent of the other. But when groups are built of two instantiations of the same individual (i.e. fraternal transition) IFF is trivially easy to satisfy and XOR is not. For equality (IFF), duplication of an individual of either type that passes through a single-cell bottleneck, or some other mechanism that enforces genetic assortment, is sufficient. But inequality (XOR) is not so simple in fraternal groups. Which is just to say that groups of heterogeneous phenotypes cannot be built from homogeneous genotypes unless there is also some mechanism of context-sensitive differentiation (e.g. plasticity).

These distinctions can also be formalised in game theoretic terms. In conventional social dilemmas, such as the Prisoner’s Dilemma, the Snowdrift game or the Stag Hunt, the social composition that maximises community welfare is 100 % cooperation (a homogeneous collective). In contrast, in *division of labour games*, the community welfare of a group is maximised by a particular combination of *multiple* strategies (Tudge et al. 2013; Tudge et al., submitted). This kind of social dilemma cannot be solved by genetic assortment alone (i.e. homogenous groups); some form of developmental process, through which particles may coordinate with one another to adopt complementary roles (West-Eberhard 2003; Jablonka 1994), is required to solve this type of dilemma (Tudge et al., submitted).

### How Evolution Changes the Evolutionary Unit

Given this view of the evolutionary challenges involved in an evolutionary transition, next we want to understand the conditions under which natural selection acting on the existing, i.e. lower, evolutionary units will meet these challenges. First we address how the action of natural selection modifies the evolutionary unit in dilemmas that can be resolved by positive assortment or homogeneous groups (e.g. the first stage of a fraternal transition). Then we address a minimal case of heterogeneous groups, i.e. two-player division of labour games, given particles with plastic phenotypes, as per a fraternal transition with minimal diversification of roles. This describes our work on *social niche construction*. For homogeneous groups, scaling-up to larger collectives is unproblematic. In contrast, for groups with multiple heterogeneous functional roles, it becomes necessary to describe the evolution of reproductive relationships in a network. Our models thus far address how natural selection modifies the evolutionary unit in networks of *genetically* heterogeneous components, i.e. egalitarian transitions. This work illustrates how the positive feedback principle, in common with the feedback in developmental and ecological networks, also applies to the evolution of reproductive relationships.

#### Social Niche Construction

Social evolution theory explains the evolution of cooperation by showing that strategy assortment makes cooperators fitter than defectors even when the reverse is true in a well-mixed population (Frank 1998; Bourke 2011). Social assortment (where similar *phenotypes* meet one another with higher probability than would be expected from their global frequency) and relatedness (where similar *genotypes* meet one another with higher probability than would be expected from their global frequency; Michod and Hamilton 1980) are exogenous parameters to this type of explanation (Ryan et al. 2015). In natural populations, however, there are many ways in which individual characters affect strategy assortment (e.g. seed dispersal radii, quorum sensing dispersal behaviours, habitat preferences, context-sensitive phenotypes) (Powers et al. 2011). We seek to endogenise parameters affecting population structures and genetic assortment into evolutionary explanations; to thereby explain the evolution of cooperation at a deeper level. For example, instead of concluding that cooperation prevails because genotypes are positively assorted, we ask why population structures that provide such genetic assortment evolved—and in particular, whether these structures evolved precisely because they enabled greater cooperation.

*Social niche construction* (Powers 2010) thus studies the concurrent evolution of social behaviours with behaviours that modify the social niche, e.g. via traits that modify population structure (Powers et al. 2011; Powers 2010; Ryan et al. 2015). This work shows various conditions under which group structure that supports cooperation can evolve under individual natural selection (Szathmáry 2011). In game theoretic terms, one way to formalise this feedback is with the concept of a *meta*-*game*, i.e. where individuals have traits that modify the game they are playing, in addition to social strategies (Jackson and Watson 2013; Jackson and Watson, submitted; Jackson 2011; Doncaster et al. 2013). This work enables us to characterise the conditions under which individual natural selection can transform social interactions to remove a social dilemma (moving the effective game from a Prisoner’s Dilemma to a Harmony Game, for example) and enabling cooperation to prevail (Jackson and Watson, submitted). This is a simple example of a feedback between a social structure and a social behaviour, echoing the other examples of feedback between an evolutionary process and the parameters of that process we have discussed. We find that natural selection can, via social niche construction, increase the level of cooperation in a population (e.g. by evolving group size in the direction that favours cooperation).

However, this does not necessarily demonstrate a new evolutionary unit that exhibits heritable variation at the collective level. This depends on whether we are modelling a type-1 or type-2 group selection process (Okasha 2006; Wilson 1989). In *type*-*1 group selection*, individual fitness is affected by a group context but groups do not have heritable lineages. Here reproduction of groups may be via an aggregation and dispersal process, where individuals reproduce via a migrant pool (Maynard Smith 1964). In *type*-*2 group selection* models, groups do have heritable lineages and fitness is thus meaningful at the group level. Here, group reproduction may be via a group-fissioning process or propagule reproduction such as in the *stochastic corrector* model (Szathmáry and Demeter 1987). In both cases, social niche construction can change the balance of particle-level and group-level selection by, for example, modifying the initial size of groups (Powers et al. 2011), or the initial size of group propagules (Ryan et al. 2015, Ryan, in prep.). But the latter is particularly relevant to the evolution of individuality at a new level of organisation because only in this case do collectives have lineages and heritable properties (Ryan et al. 2015; Ryan, in prep.). In this case (unlike the migrant pool model) it is possible to show that collectives exhibit heritable variation in reproductive success and that this increases over evolutionary time via social niche construction.

Those works have addressed conventional social dilemmas that are resolved by positive assortment (genetic and phenotypic). Other work is investigating social niche construction in models that address division of labour games and fraternal transitions. These models ask when individual natural selection (on the original evolutionary units) will utilise phenotypic plasticity, and when it will utilise context sensitive plasticity (e.g. parental effects), on the assumption that these characteristics are controlled by particle genotypes. We find that positive genetic assortment alone does not resolve such dilemmas, but positive genetic assortment is necessary to enable the evolution of negatively-assorted phenotypes via plasticity. This is because this scenario creates a new (conventional) social dilemma on genotypes between ‘co-operators’ that have the ability to coordinate phenotypes correctly, and defectors that do not. Purging such defectors is a proviso for the evolution of negative phenotypic assortment, i.e., the expression of complementary functional roles via phenotypic plasticity (Tudge et al. 2013; Tudge et al., submitted). Accordingly, positive genetic assortment creates a situation where an allele that produces coordinated phenotypic differentiation is then favoured and can thereby maximise collective fitness (Tudge et al., submitted).

Encapsulating pairs of particle-phenotypes into a new evolutionary unit causes them to be reproduced as an indivisible pair (i.e. one particle-phenotype cannot reproduce without the other if particle reproduction is appropriately policed or if the two particle phenotypes share the same genotype). Like the connections in the deep optimisation model, this changes the way combinations of particles are substituted under selection. For example, suppose a 11 pair (e.g. cooperative pair) has higher collective fitness than a 00 pair (e.g. defect pair). Before a transition, a 11 pair cannot competitively exclude a 00 pair because when particles reproduce as individuals, 01 pairs will also be created and individual selection favours 0 in this case. Put differently, before the transition the pair is not the relevant evolutionary unit. But after the transition, 11 can competitively exclude 00 when reproducing as a unit because it has higher collective fitness. Thus by eliminating fitness differences within groups, the remaining effect of selection derives entirely from between-group selection, i.e. the relevant evolutionary unit is now the pair. Putting together the ideas of social niche construction with a minimal demonstration of the between-group selection it enables, we show that individual selection creates groups that facilitate higher-level adaptation (Snowdon et al. 2011). This is analogous to the jumps in configuration space that are facilitated by the new units in deep optimisation.

These works (and others, Ghang and Nowak 2014; Szathmáry 2011) indicate that it is possible to endogenise the evolution of reproductive organisations into evolutionary theory (Ryan et al. 2015). We find that when natural selection is given the chance to do so, at least in small systems (e.g. a single pairwise relationship between two particles), it favours reproductive organisations that follow correlation learning principles—either reinforcing positive correlations or negative correlations, depending on what the social game requires. The new evolutionary units thus created can change evolutionary outcomes by creating selection that operates at the collective level rather than the particle level.

#### Scaling-up From Pairwise Relationships to Networks

In dilemmas that are solved by positive assortment, it is easy to imagine that economies of scale provide even greater benefits to larger collectives (greater than size two). But scaling-up from pairwise relationships to large numbers of particles is more challenging when it involves complementary functional roles. Intuitively, homogeneity scales easily, but heterogeneity does not—there are many ways to be different and organising these differences is, for example, what makes multi-cellular organisms interestingly different from simple colonies of cells.

In large heterogeneous systems, the relevant question changes from ‘*how* do individuals create reproductive structures that change the level of selection?’ (e.g. by social niche construction) to ‘given that individuals can create reproductive structures that change the level of selection, *which* individuals do so and *with whom*?’ Here our work focusses on egalitarian transitions where genetically diverse particles are placed together into a new reproductive unit. This work seeks to identify conditions where particle-level natural selection is successful in creating new evolutionary units that are fit given that there is variation in the membership of such units. The evolution of such units or partnerships enables individual natural selection to control which individuals reproduce independently and which are ‘in the same boat’, i.e. which individuals have shared reproductive fate/create a single vertical lineage. A particle that forms such a reproductive partnership must forgo within-collective fitness differences but may gain fitness differences at the collective level through the combinations of multiple diverse types that are enabled by these units.

Our early work in this area used a simple approach. It created higher-level evolutionary units at random and let natural selection retain those that were adaptive (Watson 2006; Watson and Pollack 2002). The *symbiotic evolutionary adaptation model* could correctly identify which composite units corresponded to sub-problems in a combinatorial optimisation problem (Watson 2006). However, this approach did not scale well. In short, there are too many possible partnerships to try and there are many partnerships that are far from optimal but fitter than not having a partnership at all. This makes it very difficult for selection to find partnerships that enable effective adaptation at higher levels of organisation. This is analogous to the problem of how supervised learning under-determines intermediate representations in deep correlation learning—there are many intermediate representations that locally improve the output but are not optimal for the higher-level task.

In hindsight, we were missing a trick; this work overlooked the value of *unsupervised* correlation learning in reducing the dimensionality of the search space before new units are created. More recent work rectifies this by exploiting principles of unsupervised learning introduced into the new approaches to deep learning. This uses individual-based simulations where, as before, individuals have traits that define symbiotic partnerships controlling who they co-disperse with during reproduction, thus creating new heritable units. But this is now combined with ecological dynamics (particle level selection) such that selection for new evolutionary units occurs mostly at local ecological equilibria. Under these conditions, new evolutionary units that join two species together must be at least as good as the combinations of species that already co-occur under individual selection at ecological equilibria—otherwise individuals that are *not* partnered will be fitter. By occasionally perturbing the ecological dynamics, we cause the system to visit many different ecological equilibria, and under these conditions, only partnerships that are robust over the distribution of ecological equilibria visited will survive selection. Accordingly, the partnerships that are favoured by selection are those that evolve to canalise the combinations of species that already co-occur most frequently under particle-level selection. This implements the unsupervised correlation learning principle, i.e. evolutionary units that reproduce together (at the same ecological equilibria) become ‘wired’ together into new evolutionary units. This greatly reduces the number of partnerships that are favoured under individual selection, and these more stringent selective conditions prove to be extremely effective in focussing selection onto partnerships that are adaptive (Watson et al. 2009a, b; Watson et al., submitted). The partnerships that evolve under these conditions subsequently enable groups of species to invade ecological equilibria as a unit, allowing the ecological dynamics to jump out of local equilibria and thereby find new ecological equilibria of higher collective fitness.

Again, this is analogous to the jumps in configuration space that are facilitated by the new units in deep optimisation. In the size-two collectives of the previous section, the computational optimisation capabilities of forming new units are not significant; assuming that there is heritable variability in the membership of egalitarian groups, forming groups at random would be sufficient to find groups that maximise collective welfare. In these larger systems, this is not the case. Forming large collectives at random is not sufficient to find collectives with high collective welfare. Moreover, incrementally modifying the membership of groups is not sufficient to find collectives with high collective welfare even when selection is enforced at the collective level (Watson et al., in prep., Watson et al. 2009a, b). In other words, finding fit collectives is a computationally difficult problem that cannot be solved by selection at the particle level nor by simply enforcing selection at the global level. However, under the selective conditions described, implementing deep optimisation with unsupervised learning principles, natural selection picks out particular subsets of species that have synergistic fitness interactions to form new units. This enables these intermediate-sized units to invade as a unit and competitively exclude other subsets of species (even if those species are not favoured under individual selection). This enables the evolving ecosystem to rescale the evolutionary process, and thereby find larger collectives with especially high collective fitness, and so on. This multi-scale process enables natural selection to find solutions that cannot be found by single-level evolutionary processes at either the individual or global level (Watson et al. 2009a, b).

#### How Does Lower-Level Selection ‘Anticipate’ Which Partnerships Will Facilitate Upper-Level Adaptation?

The reason that these partnerships evolve is not, and cannot be, because they will enable effective adaptation in future. These partnerships evolve because they are the partnerships that exhibit the immediate fitness benefits of robustness. Specifically, partnerships between particles that already frequently co-occur under particle-level selection are close to neutral with respect to their effect on collective fitness (i.e. they are partnerships between types that already co-occur). Nonetheless, it is exactly these partnerships that later enable selection at the collective level to escape local optima and discover higher fitness solutions. We can understand why this is the case by analogy with deep learning and deep optimisation: partnerships that amplify existing correlations reduce the dimensionality of the search space in a non-arbitrary way. The evolution of reproductive correlations under individual selection, shown in these models, has the effect of canalising combinations of particles that commonly co-occur at social equilibria. This occurs without any information about what combinations of particles will be adaptive at the higher level of selection that is thus created. By mimicking the structure of co-occurrence produced at ecological equilibria in this way, the evolution of new reproductive relationships is analogous to the use of unsupervised learning to reduce the dimensionality of a data set in deep optimisation. Optimisation algorithms inspired by this multi-scale process resulted in the technique of multi-scale search (Mills et al. 2014) which is able to solve formally difficult optimisation problems that provably cannot be solved by conventional (i.e. single scale) evolutionary models.

Taken together these models suggest that ‘deep evolution’, involving transitions through multiple scales of biological organisation, implements a sort of multi-scale correlation learning machine, or deep optimisation, that is quite different from the conventional micro-evolutionary (i.e. hill-climbing) model of evolutionary adaptation (Watson 2012).

## Integration

Our work illustrates several different but complementary processes by which the Darwinian Machine changes as a result of its own products. The evolution of developmental networks modifies the distribution of phenotypic variants that selection can act on, and the evolution of ecological networks modifies the selection acting on those variants.

The major evolutionary transitions involve more radical transformations in the underlying processes of the Darwinian Machine. We have illustrated several of the components that are involved in a mechanistic account of such transitions in separate models. Our models of social niche construction consider small collectives (pairwise games) and larger homogeneous collectives. Our work on large heterogeneous collectives thus far address complementary research questions by assuming that social niche construction capable of instantiating higher-level evolutionary units of genetically heterogeneous units is provided. A more integrated model should bring these works together. Our models also do not yet attempt to address fraternal transitions in large collectives of many functional roles. Further, it would be desirable to present a model where particles have the evolutionary option to employ mechanisms that either create egalitarian or fraternal transitions (or potentially a mixture of the two). This would enable us to examine the conditions under which natural selection takes different routes through the space described in Fig. 1.

From greater perspective, although the work on the evolution of developmental and ecological organisations has theoretical and conceptual unifying themes, we do not yet have a model that integrates changes in developmental, ecological and reproductive organisations freely in the same model. This would be ambitious, of course, but we believe that this is necessary to properly understand the interaction of developmental and ecological organisations in the major transitions (Fig. 1). In an egalitarian transition, the relationships between the diverse components are originally ecological relationships, i.e. between multiple species. After the transition, these ecological relationships are encapsulated within a new single unit (O’Toole et al. 2000; Bouchard and Huneman 2013), but the original functional roles and particle-level selection dynamics are retained within the new unit, at least initially. In a fraternal transition, e.g. the transition to multicellularity, the transition to a new unit occurs before functional roles diversify. Thus the functional relationships between these different types are ‘developmental’ from the outset, i.e. within a single evolutionary unit. However, such transitions create a new ‘internal selection’ ecology implemented in a new inheritance system, e.g., during ontogenesis, different cell types grow into tissues through (context sensitive) replication of *epigenetic* states (Jablonka 1994; Laland et al. 2014; Huang et al. 2005). The population dynamics of these cell populations is affected by inter-cellular interactions and competition for resources within the embryo that determine which types grow where, when and how much (Buss 1987). Accordingly, either changes in ecological organisations (capable of controlling inter-specific dependencies to enable co-existence of complementary functions), or changes in developmental organisations (capable of controlling phenotypic plasticity to coordinate differential growth of particle phenotypes with one another), are necessary components of evolutionary transitions. An integrated model of these dynamics, enabling the bottom-up evolution of new evolutionary units through successive scales of organisation, remains as the ambitious aim of this research programme.

## Conclusions

Connectionism recognises that the cleverness of cognition does not derive from the cleverness of the individual neural parts but from the organisation of the relationships between them. We have introduced the term evolutionary connectionism to recognise that, in the same way, evolutionary innovation need not originate from the adaptation of the evolutionary parts *per se* but from the evolution of the relationships between them. We have argued that this is much more than a superficial analogy between learning and evolution. Specific, but simple, organisational principles are common to correlation learning systems and the evolution of organisations. Selection for correlations that improve fitness at the system level (the evolution of developmental organisation) is equivalent to reinforcement correlation learning mechanisms. And when selection for correlations that improve fitness at the component level (the evolution of ecological organisations) act to stabilise the system state this is equivalent to unsupervised correlation learning at the system level. In an evolutionary transition, where the level of selection changes from one scale of organisation to another higher level of organisation, the ecological attractors reinforced by unsupervised learning are converted into developmental attractors of the new unit. Then, by evolving new interactions with other units at this higher level, they may enact an unsupervised correlation learning process at the next level of organisation, and so on. This deep evolution process shares algorithmic properties with deep correlation learning, and optimisation techniques based on these principles (multi-scale search, deep optimisation) have adaptive capabilities that are in a provably different class from conventional single-scale microevolution.

These connections with learning theory suggest that the special problems of each biological domain have solutions. Specifically, they suggest that;

- In evo-devo, it is not impossible for short-term selection to discover developmental organisations that facilitate future evolution. This can occur in the same way, and with the same limitations, as the ability of correlation learning to generalise from past data to perform well on previously unseen data. This requires the ability to form a model of the data (e.g. a correlation model) that captures structural regularities that are invariant over time, even if superficial structures are novel. This provides a formal basis for the evolution of evolvability.
- In evo-eco, it is not impossible for a community to be organised to reflect the structural properties of its environment (e.g. the structure of abiotic, constraints and resources in which the ecological community resides) without selection at the community level. This can occur in the same way, and with the same limitations, as the process by which unsupervised correlation learning learns the structure of a data set without performance feedback. Such an organisation arises as a consequence of selection at the level of individuals within component species, and facilitates system stability and robustness to perturbations (if the perturbations have similar structure to those experienced in the past) even though there is no notion of Darwinian fitness at the system level. This provides a formal basis for the evolution of community organisation without community selection.
- In evo-ego, it is not impossible for natural selection to favour reproductive organisations that facilitate higher-level adaptation before evolutionary units at that higher level exist. This can occur in the same way, and with the same limitations, as the way in which unsupervised learning can reduce the dimensionality of a problem in deep learning even though it has no performance metric that knows what the new representation might be used for at the next level of organisation. This implies that the evolutionary transitions may not be simply a collection of extraordinary accidents (even though each has its own unique features) but the result of systematic adaptive processes.

Although we argue that it is not impossible for biological systems to exhibit such behaviours, clearly they do not always do so. Our aim in making these connections with learning theory is so that we have potential to identify the conditions when biological systems will produce these behaviours and when they will not (Watson and Szathmáry, 2015). For example, the transfer of learning theory to evo-devo suggests that the evolution of evolvability will be sensitive to the match between the deep structural regularities of the environment and the intrinsic inductive biases of developmental processes (i.e. the kind of regularities that are easy to ‘learn’ in that ‘model space’), and to the costs and benefits of ‘overfitting’ the environment (Kounios et al., in prep., Kouvaris et al. 2015). The transfer of learning theory to evo-eco suggests that the evolution of community organisation will be sensitive to the presence of ecological constraints that cause species to coevolve dependencies with one another rather than simply evolve toward independence (Power et al. 2015). And the transfer of learning theory to evo-ego suggests that the evolution of new evolutionary units will exhibit limitations analogous to those of deep learning.

Together the evolution of developmental, ecological and reproductive organisations modifies the mechanisms of variation, selection and inheritance that drive evolution by natural selection. The evolutionary connectionism framework sheds light on how the Darwinian Machine can thereby be rescaled from one level of biological organisation to another. The results thus far demonstrate that connectionist learning principles provide a productive methodological approach to important biological questions and offer numerous new insights that expand our understanding of evolutionary processes (Watson and Szathmáry 2015). Regardless of how the exact alignment between the evolutionary and learning models discussed in this paper develops with future research, the algorithmic territory covered by learning algorithms is, we argue, the right conceptual territory for developing our understanding of how evolutionary processes change over evolutionary time (Watson and Szathmáry 2015).
